# A Tale of Three Recent Pandemics: Influenza, HIV and SARS-CoV-2

**DOI:** 10.3389/fmicb.2022.889643

**Published:** 2022-06-02

**Authors:** Mafalda N. S. Miranda, Marta Pingarilho, Victor Pimentel, Andrea Torneri, Sofia G. Seabra, Pieter J. K. Libin, Ana B. Abecasis

**Affiliations:** ^1^Global Health and Tropical Medicine (GHTM), Instituto de Higiene e Medicina Tropical/Universidade Nova de Lisboa (IHMT/UNL), Lisboa, Portugal; ^2^Artificial Intelligence Lab, Department of Computer Science, Vrije Universiteit Brussel, Brussels, Belgium; ^3^Interuniversity Institute of Biostatistics and Statistical Bioinformatics, Data Science Institute, Hasselt University, Hasselt, Belgium; ^4^Department of Microbiology and Immunology, Rega Institute for Medical Research, KU Leuven, University of Leuven, Leuven, Belgium

**Keywords:** pandemics, infectious diseases, HIV-1, influenza, SARS-CoV-2

## Abstract

Emerging infectious diseases are one of the main threats to public health, with the potential to cause a pandemic when the infectious agent manages to spread globally. The first major pandemic to appear in the 20th century was the influenza pandemic of 1918, caused by the influenza A H1N1 strain that is characterized by a high fatality rate. Another major pandemic was caused by the human immunodeficiency virus (HIV), that started early in the 20th century and remained undetected until 1981. The ongoing HIV pandemic demonstrated a high mortality and morbidity rate, with discrepant impacts in different regions around the globe. The most recent major pandemic event, is the ongoing pandemic of COVID-19, caused by the severe acute respiratory syndrome coronavirus 2 (SARS-CoV-2), which has caused over 5.7 million deaths since its emergence, 2 years ago. The aim of this work is to highlight the main determinants of the emergence, epidemic response and available countermeasures of these three pandemics, as we argue that such knowledge is paramount to prepare for the next pandemic. We analyse these pandemics’ historical and epidemiological contexts and the determinants of their emergence. Furthermore, we compare pharmaceutical and non-pharmaceutical interventions that have been used to slow down these three pandemics and zoom in on the technological advances that were made in the progress. Finally, we discuss the evolution of epidemiological modelling, that has become an essential tool to support public health policy making and discuss it in the context of these three pandemics. While these pandemics are caused by distinct viruses, that ignited in different time periods and in different regions of the globe, our work shows that many of the determinants of their emergence and countermeasures used to halt transmission were common. Therefore, it is important to further improve and optimize such approaches and adapt it to future threatening emerging infectious diseases.

## Introduction

A pandemic is caused by a newly emerging pathogen that spreads rapidly among naive human hosts, thereby affecting a great number of individuals globally (Kelly, 2011). In the last century, the most noteworthy pandemic events originated from three distinct pathogens: HIV, influenza, and SARS-CoV-2. In this review, we discuss these three pandemics from a historical, epidemiological and virological perspective, with the aim to highlight the main determinants of its emergence, epidemic response and available countermeasures. Understanding the differences and similarities between these pandemics is paramount to know where to focus on, when preparing for the next pandemic. To this end, we compare technological advances, adopted preventive measures and policy making decisions, across the distinct pandemic eras. The three pandemics discussed in this work present ideal candidates for this perspective, as they cover different time periods and correspond to different types of respiratory pathogens (i.e. influenza and SARS-CoV-2) and a sexually transmissible pathogen (i.e. HIV), which exhibit distinct virological and epidemiological characteristics. Our review demonstrates the importance of an intimate understanding of the transmission networks through which epidemics are perpetuated, and the evolution in research and technology that enable pandemic preparedness and response. Additionally, we compare the evolutionary and virological specificities of these pathogens and the impact this has on the global spread and developments of its pandemics. Furthermore, we discuss different pharmaceutical interventions, examine the revolutionary developments that took place during the HIV and SARS-CoV-2 pandemics, and project this to evaluate our preparedness for the next pandemic. Finally, we detail the importance of epidemiological models to understand the pathogen, host, and its environment, and how such models can be used to evaluate mitigation policies *in silico*. The organization of this manuscript deviates from the chronological ordering of pathogens by date of emergence, to group the respiratory pathogens (i.e. influenza and SARS-CoV-2) and to allow for a back-to-back comparison of these pathogens.

## Historical Perspective on the Emergence of Three Recent Pandemics

**Figure 1:**
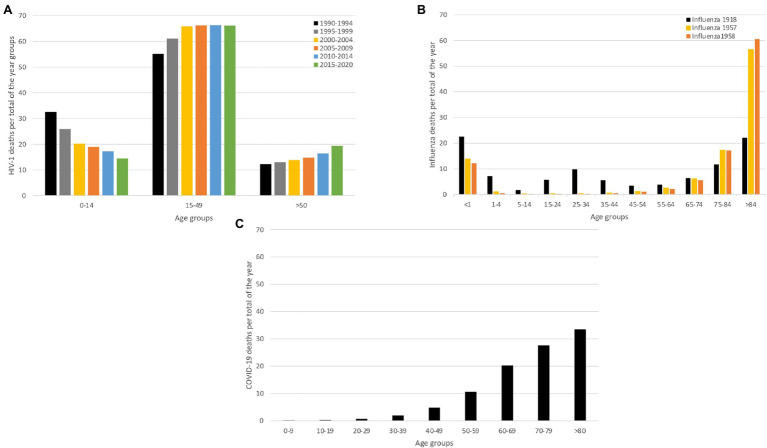
Mortality caused by HIV-1, Influenza and COVID-19. We compare mortality between two acute infectious diseases (SARS-CoV-2 and influenza pandemic) and a chronic infectious disease (HIV), to demonstrate that their impact on mortality is inherently different. For all the infectious diseases, we show deaths proportional to age (panels **A–C**). **(A)** Age-proportional deaths divided into six time-periods grouped by 4 years (1990–1994; 1995–1999; 2000–2004; 2005–2009; 2010–2014 and 2015–2020), to show the age-specific mortality evolution over the years (UNAIDS, 2022). **(B)** Influenza age-proportional deaths for three major influenza pandemics (1918, 1957 and 1958), to show age-specific mortality patterns over the different influenza pandemics (Luk et al., 2001). **(C)** Age-proportional COVID-19 deaths for the first year of the pandemic (2020), when only non-pharmaceutical interventions were available (Riffe et al., 2021). This panel demonstrates how COVID-19 deaths exponentially increase by age.

In 1981, the first cases concerning young homosexual men with depleted T-lymphocytes were reported in the United States (Greene, 2007). This depletion rendered these men susceptible to opportunistic infections, eventually resulting in death. This condition would later be known as the Acquired Immunodeficiency Syndrome (AIDS; Greene, 2007). In 1983, the cause of AIDS was yet to be identified and the number of people with AIDS in the US continued to grow (De Cock et al., 2012). It was clear that transmission occurred between individuals and the diversity of the affected individuals implied that the infectious agent could spread *via* distinct transmission routes, including sexual, vertical and blood-borne transmission (i.e. intravenous drug use and blood products; De Cock et al., 2012). To figure out which pathogen was responsible for this syndrome, scientists studied the immune response of individuals that exhibited AIDS-related symptoms, which led to the identification of a new human retrovirus in 1983, by scientists of the Pasteur institute. This retrovirus was dubbed lymphadenopathy-associated virus (LAV), as it was isolated from a patient that was diagnosed with generalized lymphadenopathy (Montagnier et al., 1984). This virus was confirmed as the cause of AIDS and was later renamed to human immunodeficiency virus (HIV; Gallo and Montagnier, 2003). By then, the HIV pandemic had already unfolded, with high numbers of infections worldwide, resulting in high morbidity and mortality in the affected patients across different age groups until recent years as shown in Figure 1A.

**Figure 2:**
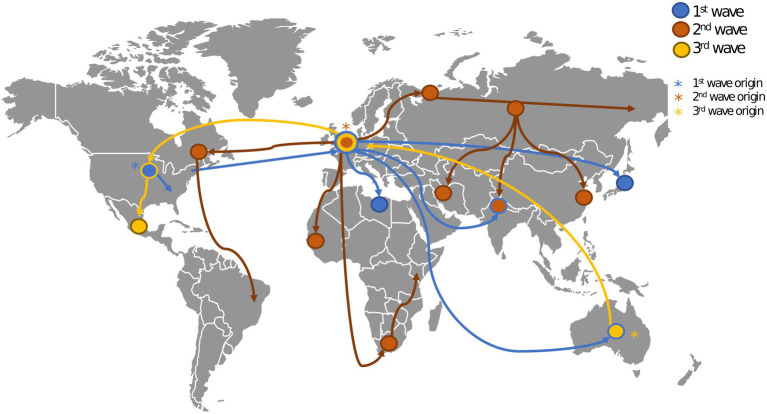
Geospatial spread of influenza during the 1918 pandemic. This figure shows the origin (marked with asterisks) and spread (marked with edges) of the three waves of the 1918 influenza pandemic (Earth.Org, 2022). The first wave originated in the United States (US). and spread throughout Europe and the rest of the world. The second wave originated in Europe and spread through the US, Europe, Asia and Africa. The third wave originated in Australia and spread through Europe and the US.

The 1918 Influenza pandemic, colloquially referred to as the ‘Spanish flu’, was one of the most devastating pandemics of the 20th century. During this pandemic, three waves were recorded, with the first cases reported in March 1918, as show in Figure 2. The pandemic caused infections in one third of the world population and it is estimated that it caused between 50 and 100 million deaths worldwide (Morens and Fauci, 2007; Spreeuwenberg et al., 2018). The virus was first reported in Kansas (United States of America) and was spread from there throughout Europe and the rest of the world by American military personnel (Martini et al., 2019). This pathogen induced illness in all age groups, but healthy young adults accounted for half of the influenza-related deaths, which is quite atypical for influenza epidemics, and this gave the death rate versus age figure a unique W-shaped pattern as shown in Figure 1B (Taubenberger and Morens, 2006). In 1933, more than a decade after the end of this pandemic, the causative influenza virus was isolated (Barberis et al., 2016). After the 1918 pandemic, influenza viruses continued to circulate globally, with new and re-emerged strains, causing other pandemic outbreaks [including the 2009 influenza pandemic (Smith et al., 2009)] and contributing to seasonal flu epidemics (Liu et al., 2009; Nickol and Kindrachuk, 2019).

The *Coronaviridae* family receives its name after the virus’ crown-like morphology. In 1965, a human coronavirus (strain B814) was isolated for the first time (Liu et al., 2021). It was generally believed that coronaviruses typically led to mild symptoms in humans (Liu et al., 2021). This however changed in 2003, when a new human coronavirus, identified as SARS-CoV, emerged in China and caused a pandemic (Weiss and Navas-Martin, 2005). This global epidemic ended through the eradication of SARS-CoV by the use of infection control measures, such as contact tracing and effective quarantine (Baric, 2008). In December 2019, an outbreak of pneumonia in Wuhan, Hubei Province in China was reported to WHO. The cause of the outbreak was identified to be a novel coronavirus related to SARS-CoV that was named SARS-CoV-2. This virus manifests in distinct clinical outcomes, ranging from asymptomatic infection to COVID-19 disease, with a mild to severe (and potentially fatal) progression (Wang et al., 2020). This virus caused a pandemic that is still ongoing, and by February 2022, there were 383 million recorded cases and 5 million deaths confirmed globally (WHO, 2022a). Figure 1C represents the year only the deaths of 2020.

These pandemics all contributed to the development of public health measures, in distinct scientific eras. The 1918 influenza pandemic took place at a time when knowledge about viruses and their transmission dynamics was scarce. The HIV pandemic started more recently when knowledge and investigation of viral diseases were growing. At that time, antiviral drugs aimed at controlling viral load in infected individuals were developed quite rapidly. For SARS-CoV-2, scientific insights were produced at an unseen pace. For example, the viral strain of the index case was isolated and sequenced as soon as 11 January 2020. Furthermore, the scientific process benefited from many useful tools for virus investigation such as molecular epidemiology and next generation sequencing (NGS), novel diagnostic tools, epidemiological models to evaluate preventive measures and an unprecedented race towards the development of vaccines and treatment options. While such advances are both impressive and encouraging, many of the preventive measures used at the start of the SARS-CoV-2 pandemic were similar to those used during the 1918 influenza pandemic, where the focus was predominantly on non-pharmaceutical interventions.

## Determinants of Emerging Diseases

We will discuss the major determinants of HIV, pandemic influenza, and SARS-CoV-2, focusing on the host, the infectious agent and the environment in which they interact.

The infectious agent is the focal point of any emerging disease. There are a variety of infectious agents, including viruses, fungi and bacteria. Such agents cause infectious diseases by infecting hosts and the agents’ properties will determine their potential to adapt to new hosts and environments. For a virus, these properties include the mutation rate, the cell tropism, the virus’ antigenic immunodominance and the virus’ ability to escape innate immune responses (Morens and Fauci, 2020). Such properties will determine the virulence and infectiousness, which might evolve whilst an epidemic unfolds (Morens and Fauci, 2020).

The host constitutes the organism that is susceptible to infection by the infectious agent; in this work, we consider viruses that can infect animals and humans. Once the host becomes exposed to a pathogen, viral replication can be initiated if the host is capable of expressing surface proteins or cellular receptors to which the virus can bind to (McArthur, 2019). In order to facilitate the ability for viruses to infect new host species, the virus needs to adapt to this new host, which is referred to as a host-switching event or zoonotic jump (Morens and Fauci, 2020). Transmission and pathogenesis can also be impacted by behavioural factors of the host and therefore transmission routes, clinical outcomes and symptoms can be altered in the new host species (McArthur, 2019; Morens and Fauci, 2020).

The environment constitutes the external conditions that affect the host that enable the emergence and persistence of a pathogen. Examples of such conditions are exposure to wildlife, environmental degradation, malnourishment and atmospheric conditions (Chen et al., 2021). Next to the impact on emergence, environmental factors can also impact the further spread of viruses.

We will now discuss how these determinants acted specifically for the emergence of the viruses discussed in this work: HIV, pandemic influenza, and SARS-CoV-2.

**Figure 3:**
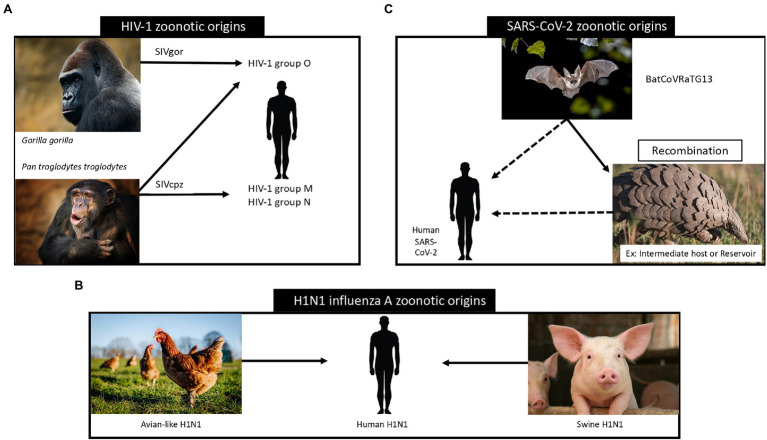
Zoonotic origins of HIV-1 (panel **A**; Tebit and Arts, 2011), influenza (panel **B**; Taubenberger and Morens, 2006) and SARS-CoV-2 (panel **C**; Konda et al., 2020) viruses and their relation to human transmission. In panel **A** we show the zoonotic transmission of Simian immunodeficiency viruses (SIV) from non-human primates to humans, leading to the development of the human immunodeficiency virus. Panel **B** depicts the main zoonotic origins of influenza A H1N1 viruses and their transmission to human hosts. In panel **C** we visualize the assumed zoonotic origin of SARS-CoV-2 virus.

**Figure 4:**
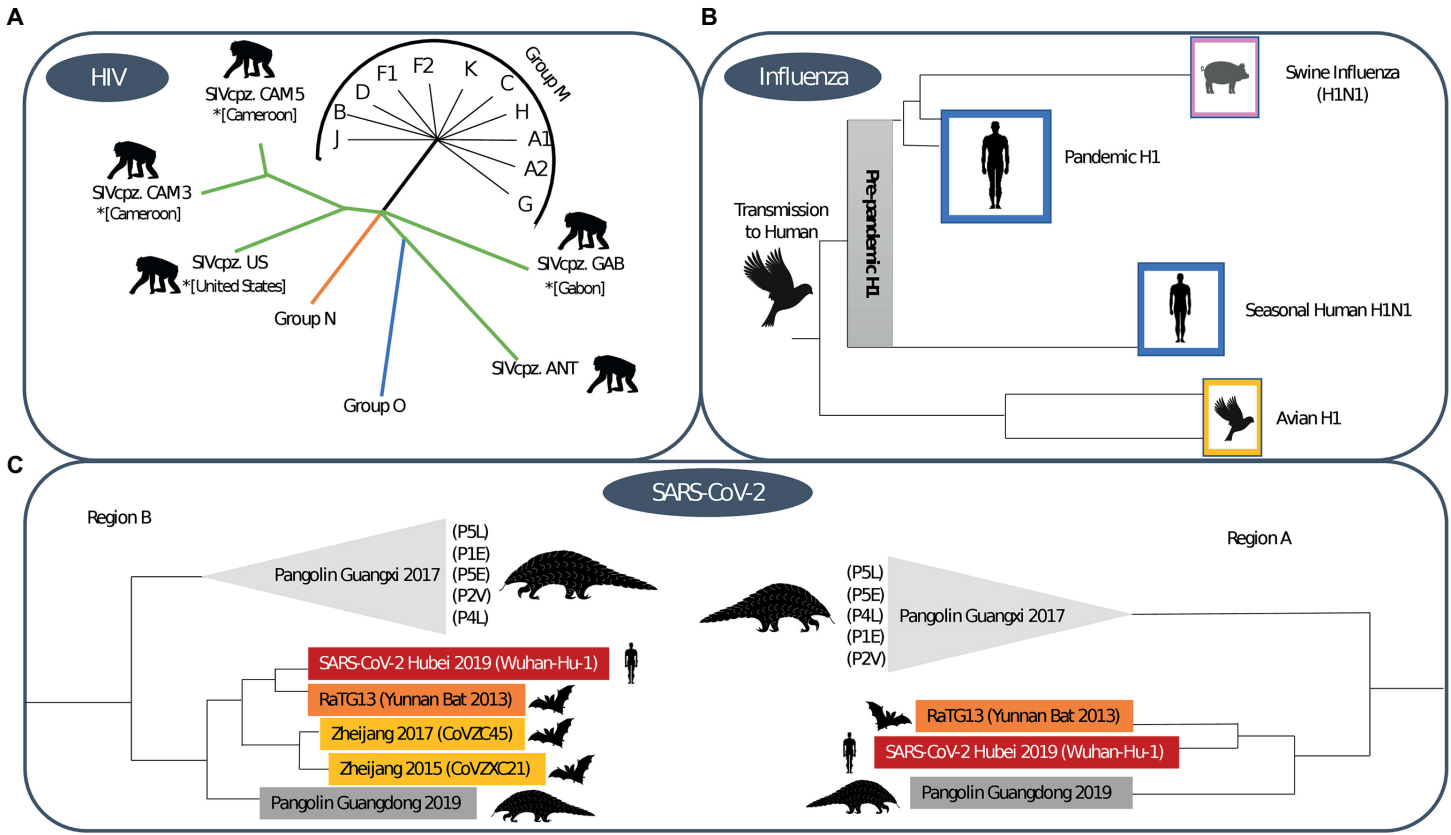
Phylogenetic trees for **(A)** HIV-1, **(B)** Influenza and **(C)** SARS-CoV-2. The HIV-1 phylogenetic tree shows sequences that demonstrate the zoonotic jump of the distinct HIV-1 groups (Thomson et al., 2002). The influenza phylogenetic tree was based on the H1 subtype, the initial origin of the 1918 pandemic and the post-pandemic spread through human and animal species (Xu et al., 2008). The SARS-CoV-2 phylogenetic tree shows the origin of sarbecovirus and is based on distinct regions, where region A is shorter due to the potential of recombination of the genome region and region B is wider (Boni et al., 2020).

**Figure 5:**
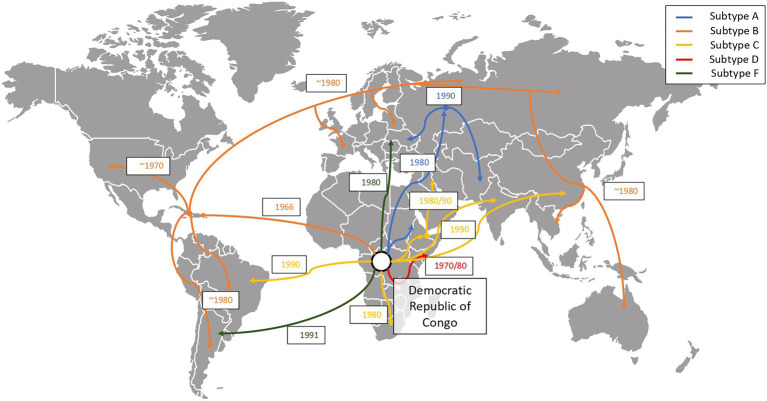
Global evolution and chronology of events of spread of the HIV-1 group M subtypes (Tebit and Arts, 2011). We show how HIV-1 group M disseminated from its original epidemic location (Kinshasa, Democratic Republic of Congo) to other regions of the globe. The figure depicts how subtype B is the most widely spread subtype, subtype A spread mostly to the east regions of Africa, Europe and Asia and subtype C spread mostly through Brazil, South Africa and Southeast Asia.

Simian immunodeficiency viruses (SIV) were transmitted from non-human primates to humans leading to the development of the human immunodeficiency virus (HIV). In Figure 3A, we show how these zoonotic events led to the HIV pandemic. HIV is a single stranded RNA virus that is part of the *Retroviridae* family and Lentivirus genus (Requejo, 2006). In the early 20th century, at least four independent zoonotic transmission events, originating from these primates, led to the origin of the four currently described HIV-1 groups. Group M, responsible for the global HIV-1 pandemic, directly originated from the chimpanzee *Pan troglodytes troglodytes* (Korber et al., 2000), as show in Figure 4A. The scientific consensus on the genesis of HIV-1 group M is that the zoonotic transmission event took place in Central Africa, possibly through hunting chimpanzees for bushmeat. The epidemic ignition, on the other hand, occurred in Kinshasa (Democratic Republic of Congo) likely due to increasing mobility of populations that occurred in that area during the first half of the 20th century (Faria et al., 2014). From there it spread to other regions in Africa and subsequently different strains were exported regions throughout the world (Korber et al., 2000), as visualised in Figure 5.

HIV-1 group M exhibits a great amount of natural diversity, with an evolutionary process that is driven both by within-host and between-host dynamics (Theys et al., 2018). This virus can generate 9 × 10^−5^ mutations per nucleotide per virus replication cycle (Hemelaar, 2012), and is prone to recombination events. The resulting viral diversity can be classified in nine subtypes and a set of Circulating Recombinant Forms (CRFs; Burke, 1997; Hemelaar, 2012). The genetic diversity that is exhibited by HIV, resulted in numerous sub-epidemics, heterogeneous in nature, and influenced by patterns of human migration and globalization (Vandamme et al., 2011; Pimentel et al., 2020). While the HIV-1 pandemic progressed, the extraction of genetic sequences became more accessible, resulting in the availability of a large collection of HIV-1 strains (Stoesser et al., 2004). Using these sequences, the subtype and CRF nomenclature enable the studying of this global spread (Alcantara et al., 2009; Pineda-Peña et al., 2013; Hemelaar et al., 2019). Furthermore, we note that these subtypes are associated with differences regarding transmissibility (Alcantara et al., 2009), susceptibility to antiretroviral drugs (Ngcapu et al., 2017), pathogenicity (Hemelaar et al., 2019) and preferred route of transmission (van Harmelen et al., 1997). HIV viruses are typically transmitted through the exchange of bodily fluids, such as blood, pre-ejaculate, semen and vaginal fluids. Once an infection has been established, the HIV virus induces a chronic infection that can be broadly categorized in three stages (Enger et al., 1996). First, about 2–4 weeks post HIV infection, the individual will experience an acute stage that is associated with flu-like symptoms. While this acute stage typically only lasts a few weeks, due to the high viral load (and infectiousness) that is associated with this phase, infected individuals have the potential to generate many new infections, depending on their behavioural context (HIV.gov, 2021). This highlights the importance of frequent testing of high-risk individuals and raising awareness of flu-like symptoms in the context of an early HIV infection. Second, the infected individual enters a stage where the viral load drops steeply, and the virus will multiply at a lower rate. At this point, infected individuals will typically no longer experience any symptoms. When the infection progresses without antiretroviral treatment, this stage can last between 10 and 15 years. During this stage, the individual’s infectiousness is lower and depends on the patients’ set-point viral load. Due to the long period during which individuals are not aware of their infection state, there is the potential to generate a large number of infections inadvertently (HIV.gov, 2021; CDC, 2021a). Therefore, shortening the time-to-diagnosis is key to reduce HIV incidence (CDC, 2021a). Furthermore, early diagnosis decreases the onward transmission of the virus and increases the chances of treatment success (Miranda et al., 2021). Over this period, the individual will slowly progress into the third stage, at which the viral load will rise again, resulting in a rapid decline in CD4 count, which preludes the third and final stage, that is, the AIDS stage. During this phase, the immune system of the patient is weakened, which results in a wide range of opportunistic diseases, eventually resulting in death (HIV.gov, 2021; CDC, 2021a). By 2020, 36.3 million people had died from AIDS-related disease worldwide, since the start of the pandemic (UNAIDS, 2021).

HIV is transmitted *via* different routes, the most common being sexual contacts, both *via* vaginal and anal intercourse (Moir et al., 2011). Another major route of transmission is *via* intravenous drug use, blood products and vertical transmission (Moir et al., 2011). The probability for a transmission to take place depends both on the transmission route and on the viral load in the exchanged body fluids (Galvin and Cohen, 2004). Furthermore, the occurrence of other sexually transmitted diseases are associated with an increase in the risk of sexual transmission of HIV (Galvin and Cohen, 2004). The transmission route of HIV in different regions of the globe is dynamically influenced by cultural and societal factors. For example, while in Africa, the main route of transmission is *via* unprotected heterosexual contact followed by mother-to-child transmission (Fettig et al., 2014), in Latin and Central America new cases are predominantly generated between homosexual men (MSM) and *via* the sharing of needles by intravenous drug users (IDU; Fettig et al., 2014). In North America and Western Europe, the main transmission route is sexual, where MSM are most at risk (Van De Vijver and Boucher, 2018). In Eastern Europe and Central Asia, the most prevalent mode of transmission remains *via* IDU (Fettig et al., 2014; Paraschiv et al., 2017).

Influenza viruses are part of the *Orthomyxoviridae* family and *Alpha influenza* virus genus (Taubenberger et al., 2019) and have been circulating among humans since time immemorial (Nickol and Kindrachuk, 2019). Influenza viruses constitute a group of single-stranded RNA viruses, of which currently, there are four types circulating, with type A being the most widespread. They are divided into subtypes according to the type of proteins that exist on their surface, considering both the type of hemagglutinin (H) and neuraminidase (N; Xu et al., 2008). The virus that caused the 1918 pandemic originated through recombination between the H1 and N1 subtype, and is thus referred to as Influenza A (H1N1; Humphreys, 2018). We visualise the phylogenetic tree that supports this zoonotic origin in Figure 4B.

The 1918 pandemic was caused by an influenza A virus, which originated from natural reservoirs of birds, specifically waterfowls and shorebirds (Taubenberger et al., 2019), as shown schematically in Figure 3B. An important catalyst of the 1918 influenza was the first world war and its aftermath, forcing individuals to live close to each other, and thus facilitating the spread of this respiratory virus (Humphreys, 2018).

For the 1918 influenza virus, it was shown that there was a significant variability in transmissibility (Fraser et al., 2011). While there was no certainty on transmission routes during the 1918 pandemic (Taubenberger and Morens, 2020), later the main respiratory transmission pathway of influenza (i.e. *via* droplets or aerosols) was demonstrated in animals (Mermel, 2018), in combination with physical contact and fomite contact (Brankston et al., 2007). Certain factors were considered to delay or enhance the transmission of the virus As with HIV, the increasing mobility of populations had an important role in the dissemination of the pandemics: as an example, the migration of soldiers from war fronts in crowded trains likely enhanced the spread of the virus (Martini et al., 2019). With respect to the transmission dynamics, it was reported that individuals were infectious prior to symptoms onset (Mills et al., 2004).

Coronaviruses (CoV), a kind of single-stranded RNA viruses, have been circulating in animals, such as birds and mammals, since the dawn of time and make up the *Coronaviridae* family of the *Betacoronavirus* genus (Malik, 2020). Bats are assumed to be a major CoV reservoir and phylogenetic analyses indicate that the origin of SARS-CoV also lies in this reservoir (Konda et al., 2020). It remains unclear whether SARS-CoV emerged due to a zoonotic event involving bats, as racoon dogs, pangolins and ferret badgers were also identified as intermediate hosts, as depicted in Figure 3C (Konda et al., 2020). In 2012, the Middle East Respiratory Syndrome coronavirus (MERS-CoV) emerged in Saudi Arabia (Umakanthan et al., 2020), and it is also believed that MERS-CoV also finds its origin in bat reservoirs (WHO, 2021a). For MERS-CoV goats, sheep and cows and the Arabian dromedary were identified as intermediate hosts (Umakanthan et al., 2020). In 2019, SARS-CoV-2 was identified and shown to be phylogenetically related to SARS-CoV (Umakanthan et al., 2020). SARS-CoV-2 is thought to have originated through recombination between a bat CoV and a CoV of unknown origin (Umakanthan et al., 2020). We show the phylogenetic tree that supports this origin hypothesis in Figure 4C.

While SARS-CoV-2 is the causative agent of COVID-19, a disease that can be mild or severe, many infected individuals remain asymptomatic. As the risk of developing severe disease grows exponentially with age, as shown in Figure 1C, we note that children are likely to be asymptomatic (Palmer et al., 2021). When suffering from severe COVID-19, the respiratory and immune system are fragile and the development of opportunistic diseases like pneumonia can occur (WHO, 2021b). Furthermore, severe COVID-19 can induce acute respiratory distress syndrome (ARDS) and other potentially fatal complications (Xu et al., 2020). Besides severe COVID-19, in some patients the virus can cause long-term illness, commonly referred to as Long-COVID, where individuals continue to experience symptoms for several months after being infected (Harrison et al., 2020). The large proportion of SARS-CoV-2 cases that results in an asymptomatic or very mild progression (Sekine et al., 2020; comparative to the common cold), that complicates contact tracing efforts, and facilitates the rapid spread of the virus (Davis et al., 2021). Furthermore, pre-symptomatic infection is an important driver of SARS-CoV-2 transmission, which renders a symptom-based isolation approach less effective. SARS-CoV-2’s main transmission route is *via* respiratory droplets (Umakanthan et al., 2020) and the virus is also considered capable of airborne transmission in ill-ventilated environments (Prather et al., 2020). At the start of the pandemic fomite contacts were hypothesized to play an important role in the transmission of SARS-CoV-2, however, recent studies indicate that transmission *via* contact surfaces is limited (Lotfi et al., 2020; Umakanthan et al., 2020). The infectiousness of SARS-CoV-2 has been shown to be highly heterogeneous indicating that the effects of superspreading should be carefully considered when drafting control measures, usually these control measure are based on local measures where it is easier to control the SARS-CoV-2 spread (Sneppen et al., 2021). While a pandemic is inherently a global process, containment strategies are to be implemented in a local setting. Therefore, understanding local transmission dynamics is vital to optimize epidemic control, which is demonstrated in the spatial analysis of SARS-CoV-2 spread in New York City (Dellicour et al., 2021).

SARS-CoV-2 has been circulating since the end of 2019 and over the course of the pandemic new variants evolved. Individuals can be diagnosed *via* polymerase chain reaction PCR or lateral flow antigen (Ag) testing (Peto et al., 2021). To monitor the evolution of SARS-CoV-2, a proportion of positive samples can be rapidly sequenced using next-generation sequencing technologies (Chiara et al., 2021). Through these sequences, and with the development of computational tools to process such large amounts of sequences (Hufsky et al., 2021), the evolution of SARS-CoV-2 was monitored in real time (Oude Munnink et al., 2021). To propose a system to classify SARS-CoV-2 lineages, the nomenclature implemented in the Pangolin COVID-19 lineage assigner^1^ was introduced (O’Toole et al., 2021). The first mutation to be detected was D614G, which increased transmissibility and infectiousness and replaced the original SARS-CoV-2 strain becoming the dominant form of the virus circulating worldwide (Zhou et al., 2021). This event was followed by the emergence of a series of variants of concern (VoC) and a variant of interest (VOI) as listed in Table 1. The Omicron variant is the most recent VoC (WHO, 2021c).

**Table 1:** Description of variants of concern and variant of interest (CDC, 2021d; WHO, 2021c,d; European Centre for Disease Prevention and Control, 2022).

Pangolin nomenclature	WHO nomenclature	Spike mutations of interest	Main properties	Country of first detection	Date classified as VOC/VOI	Date classified as previous VOC/VOI
VOC B.1.1.7	Alpha (VOC)	N501Y, D614G, P681H	➔ Increased transmissibility and severity	United Kingdom	December 2020	March 2022
VOC B.1.351	Beta (VOC)	K417N, E484K, N501Y, D614G, A701V	➔ Increased transmissibility and severity, immune escape	South Africa	December 2020	March 2022
P.1	Gamma (VOC)	K417T, E484K, N501Y, D614G, H655Y	➔ Increased transmissibility and severity, immune escape	Japan	January 2021	March 2022
B.1.617.2	Delta (VOC)	L452R, T478K, D614G, P681R	➔ Increased transmissibility and severity, immune escape	India	May 2021	
B.1.1.529	Omicron (VOC)	A67V, Δ69-70, T95I, G142D, Δ143-145, N211I, Δ212, ins215EPE, G339D, S371L, S373P, S375F, K417N, N440K, G446S, S477N, T478K, E484A, Q493R, G496S, Q498R, N501Y, Y505H, T547K, D614G, H655Y, N679K, P681H, N764K, D796Y, N856K, Q954H, N969K, L981F	➔ Large number of mutations; ➔ Increased transmissibility, reduced severity, immune escape	South Africa	November 2021	
B.1.621	Mu (VOI)	R346K, E484K, N501Y, D614G, P681H	➔ Increased impact on transmissibility ➔ Increased impact on immunity	Colombia	August 2021	March 2022

While the 1918 influenza pandemic and SARS-CoV-2 pandemic emerged in different eras, both epidemics had to rely on similar non-pharmaceutical measures. Nonetheless, through technological advances, antiviral drugs have been developed at an unprecedented pace, both for HIV and SARS-CoV-2. Furthermore, the introduction of novel vaccine platforms, such as mRNA and vector-based vaccines, have led to an unprecedented race towards vaccines in the SARS-CoV-2 pandemic.

One major similarity between these three viruses is that transmission from asymptomatic patients plays a major role in viral dispersion. As such, asymptomatic individuals continue to spread the viruses inadvertently, as they are unaware of their infection status. In that context, early diagnosis through testing is an important strategy to contain transmission. We therefore stress the importance of the availability of efficient diagnostics, such that infected individuals can take the appropriate precautions is vital to control an epidemic. PCR and Ag rapid testing strategies grown unprecedently in the context of the COVID-19 pandemic. We believe that this will be an important venue to prepare for future outbreaks.

Furthermore, all these three pandemics, as most others find their origin on zoonotic events, indicating that monitoring zoonotic spill over events is another crucial research field (Allen et al., 2017). In this context, finding potentially threatening Emerging Infectious Diseases implies intensifying research in a One Health perspective and not only at the human level (McArthur, 2019). The movement and expansion of zoonotic reservoirs throughout different territories, due to the quick environmental evolution, for example due to climate change or urbanization, also warrant special attention (Baker et al., 2021).

## Pharmaceutical and Non-pharmaceutical Interventions

### Non-pharmaceutical Interventions

The prevention strategies for HIV-1 have historically changed and evolved over the last 40 years of the pandemic. These have included sexual abstinence, condom use, promoting the use of sterilized needles for intravenous drug users (IDUs), and improved protocols to decrease the diagnosis delay for newly infected individuals (Alnwick et al., 2007). In low-income countries, the use of male circumcision has also shown to be an effective prevention strategy (Williams et al., 2006). In recent years, treatment as prevention (TasP) has become a crucial prevention approach to halt the HIV-1 pandemic, either through pre-exposure prophylaxis (PrEP), post-exposure prophylaxis (PEP) or through treatment for all.

When implementing prevention strategies it is vital to target vulnerable populations, but due to stigma and discrimination, this remains challenging (Alnwick et al., 2007). Furthermore, due to particularities in the cultural and societal context of the affected region (Wegbreit et al., 2006), prevention strategies need to be tailored to the relevant context (Wegbreit et al., 2006; Pineda-Peña et al., 2019).

When the H1N1 pandemic erupted in 1918, non-pharmaceutical interventions were the only available resources to control the epidemic. These interventions include the notification of suspected cases to authorities, the isolation of infected individuals and a mandated quarantine for the contacts of infected individuals (Markel et al., 2007; Martini et al., 2019). Furthermore, the use of face masks, surveillance of communities, closure of meeting venues and hygienic measures were implemented to reduce transmissions (Markel et al., 2007; Humphreys, 2018; Martini et al., 2019).

For SARS-CoV-2, individuals are isolated at home when they experience any COVID-19-related symptoms, until they obtain a negative test or recover from the infection (Pradhan et al., 2020). Furthermore, as individuals are highly infectious prior to developing symptoms and as many infected individuals stay asymptomatic or with very mild symptoms that can be easily mistaken for a common cold, additional prevention measures are necessary (Pradhan et al., 2020). Such prevention measures include early screening or self-testing and diagnosis (through testing and contact tracing), isolation, social distancing, facial masks and hygienic measures (e.g. hand washing, alcohol-based sanitizer, cleaning surface environments; Pradhan et al., 2020). Given that SARS-CoV-2 is transmitted *via* the respiratory tract (i.e. droplets or aerosols), the use of masks and ventilation is particularly important (Peeples, 2021). However, different surges globally have demonstrated that these measures can easily fail when social distancing measures are released and the use of mass testing has been suggested to control the epidemic (Burki, 2020).

### Antiviral Drugs for Treatment and Prevention

As a cure for HIV remains elusive to date, highly active antiretroviral therapy (HAART) is the only option to improve the quality of life of an HIV patient (Cohen et al., 2008). Upon HAART, patients achieve viral suppression to increase the CD4 cell count and recover the immune system function, which improves the clinical status of the patient (Pau and George, 2014). Furthermore, contemporary HAART regimens decrease viral load to undetectable levels, thereby significantly reducing the infectiousness of the treated individual (Pau and George, 2014). This observation led to the use of antiviral therapy to prevent individuals from getting infected, so-called Pre-exposure prophylaxis (PrEP), which is mostly used in people at increased risk of contracting HIV. PrEP has been demonstrated effective in reducing the risk of HIV-1 infection (Alnwick et al., 2007; Cohen et al., 2008; Fonner et al., 2016).

The development of HAART introduced a revolutionary improvement for HIV patients. However, due to the fast evolutionary rate of HIV and the selective pressure that is induced by HAART, resistance mutations to antiretroviral drugs can emerge and impede viral suppression (Pingarilho et al., 2020). The resistance to antiretroviral drugs can manifest in two ways, as a result of selective pressure of antiretrovirals in treated individuals (i.e. acquired drug resistance) and as a result of an infection with a virus strain that carries drug resistance mutations (i.e. transmitted drug resistance; Baesi et al., 2014; Clutter et al., 2016). Drug resistance testing is a necessary tool to detect transmitted drug resistance in newly diagnosed patients in order to guide the selection of antiretroviral therapy, to minimize the risks of virological failure (Charles and Hicks, 2008). Resistance to antiretroviral therapy, that could be related to poor treatment adherence, is still a global reality and a major barrier to end HIV/AIDS pandemic (Kim et al., 2018). A new approach that can minimize problems of adherence is the use of injectable long-acting HIV medication, which has been shown to be well accepted and effective (Simoni et al., 2020). Also, it is important to mention that contemporary antiretrovirals have a higher genetic barrier, meaning that the virus is less likely to escape from selective pressure (Theys et al., 2019).

For influenza, the use of antiviral drugs can be used to reduce morbidity and mortality, and as a prophylactic agent to reduce individual susceptibility. Nonetheless, when a novel influenza strain emerges, it remains unclear whether resistance mutations could hinder the antiviral activity of the current generation of antiviral drugs (Lipsitch et al., 2007; Wang et al., 2013; Hussain et al., 2017; Lee and Hurt, 2018). There are three groups of therapeutic drugs for influenza: the neuraminidase inhibitors, the M2 inhibitors and the polymerase inhibitors (De Clercq, 2006). The neuraminidase inhibitors include oseltamivir, which is the most used and its efficacy is proved to prevent transmission and outbreaks within households (Welliver et al., 2001), zanamivir and peramivir (Lee and Hurt, 2018; Chow et al., 2019). The class of M2 inhibitors includes amantadine and rimantadine (De Clercq, 2006; Keshavarz et al., 2020). A novel therapeutic for influenza virus is Baloxavir marboxil: a cap-dependent endonuclease inhibitor that inhibits viral mRNA synthesis (Du et al., 2020; Lampejo, 2020). The most common resistance to influenza antivirals is towards M2 inhibitors, which is the major reason why this class is no longer widely used (Wang et al., 2013). Resistance to neuraminidase inhibitors have also been reported and further monitoring is warranted (Lee and Hurt, 2018).

For SARS-CoV-2, besides vaccines, the use of monoclonal antibodies, interferon-based preventive therapies and antiviral agents have been considered (Lotfi et al., 2020). Interferon-α and interferon-β were previously approved for, respectively, hepatitis B and for chronic obstructive pulmonary disorder. These interferons are now under investigation for SARS-CoV-2 prevention (Ahsan et al., 2020). Currently, different studies for the use of monoclonal antibodies are ongoing (Valle et al., 2020; Malani and Golub, 2021; Starr et al., 2021), yet the potential development of resistance mutations poses a threat to the efficacy of such therapeutics. Different antiviral agents have been considered, both as prophylactic and therapeutic agents, including drugs previously targeted at HIV-1 (Andreani et al., 2020; Lotfi et al., 2020) or Ebola (i.e. remdesivir; Kumar et al., 2020). However, these regimens were disappointing in clinical trials, and only recently a set of antiviral drugs has shown promise. As of December 2021, the United States Food and Drug Administration approved Paxlovid (a combination of nirmatrelvir and ritonavir) for the treatment of mild-to-moderate COVID-19 disease (U.S. Food and Drug Administration, 2022), although other antiviral drugs are currently being considered for approval, i.e. molnupiravir and an orally active 3CL protease inhibitor (Boras et al., 2021; GOV.UK, 2021).

### Vaccines

Currently, there is no vaccine for HIV-1, with many trials rendering unsatisfying results (Tebas et al., 2012; Pollard et al., 2014; Ensoli et al., 2015; Dinges et al., 2016; Macatangay et al., 2016; Jacobson et al., 2016a,b; Brekke et al., 2017), which is often attributed to the extensive genetic diversity that is exhibited by HIV-1. Recent work investigates the use of mRNA-based vaccines that incorporate multiple HIV subtypes, in order to cover a larger genetic diversity (Aidsmap, 2021).

Since 1918, there has been immense progress with respect to influenza vaccine development, and these technologies have been used to control more recent influenza pandemics and seasonal influenza epidemics (Wu et al., 2010; Huddleston et al., 2020). Vaccines have shown to decrease hospitalizations, susceptibility and infectiousness (Koszalka et al., 2017; Robson et al., 2019). Yet, in case of a pandemic emergency, it will take time in order to produce vaccines for a novel influenza strain, rendering the use of non-pharmaceutical intervention measures necessary until the vaccine becomes widely available (WHO, 2004).

For Influenza A there are two types of vaccine platforms available, the inactivated influenza vaccines and the life attenuated influenza vaccines. The viruses for inactivated vaccines can be grown in eggs, cell culture or using recombinant technology (WHO/Europe, 2021; CDC, 2021b). The life attenuated influenza vaccine consists of a nasal spray vaccine (WHO/Europe, 2021; CDC, 2021b). Also, studies are ongoing to use mRNA technology as a new platform for influenza vaccines (Moderna, Inc., 2021).

For SARS-CoV-2, vaccines were developed at an unprecedented pace. A large number of vaccine platforms has been studied in the context of SARS-CoV-2, but the class of mRNA vaccines and vector-based vaccines were the first to show promising results (Bettini and Locci, 2021; FDA, 2021; CDC, 2021c). The most common class of vaccines, available since late 1800s, are the inactivated vaccines derived from killed virus, which also have been produced for COVID-19. However, this type of vaccine had a time and complexity of production higher when compared to mRNA and vector-based vaccines (Plotkin, 2014; Park et al., 2021; Zhang et al., 2022). These latter two vaccine classes were most widely used during the COVID-19 pandemic. The mRNA vaccine uses a piece of mRNA to instruct the production of a specific protein that will be recognized by the human immune system. While the development of mRNA vaccines has been active for at least 30 years (Dolgin, 2021), this class of vaccines has been approved for human use for the first time during the COVID-19 pandemic (WHO, 2022b). The vector-based vaccines, studied since 1982 (Ura et al., 2014) and first approved in 2019 to prevent Ebola virus (Vrba et al., 2020), aim to trigger an immune response by delivering a specific protein of the pathogen (Creech et al., 2021; WHO, 2022b).

When these vaccines were first released, they were welcomed with great optimism given their high effectiveness, both regarding preventing symptomatic infection (VE_I_) and severe disease (VE_S_). This was the case for the vector-based vaccines, where a single dose of the Janssen vaccine showed a VE_I_ of 85% (Andreano and Rappuoli, 2021), while for the Vaxzevria vaccine (two doses) a VE_I_ of 73% was recorded (Lopez Bernal et al., 2021). Equally so for the mRNA-based vaccines, where the Pfizer-BioNtech vaccine (two doses) was reported a VE_I_ of 91.1% according to a study of safety and efficacy through 6 months (Thomas et al., 2021), while the Moderna vaccine (two doses) displayed a VE_I_ of 94.1% (WHO, 2022c). When new variants emerged, the VE_I_ was reduced, yet, for individuals that were administered a two-dose regimen, VE_S_ remained high (Tregoning et al., 2021). The wide distribution of vaccines therefore significantly reduced hospital loads, even after the emergence of the first variants (Lauring et al., 2022). For the most recent Omicron VoC, to this point the most infectious variant, the two-dose regimen shows a significant reduction in both VE_I_ and VE_S_ (Andrews et al., 2022). However, recent research shows that a third vaccine dose boosts vaccine efficacy both against symptomatic infection and sever disease (Andrews et al., 2022; Nyberg et al., 2022).

Since December 2020, three vaccines have been approved by the United States Food and Drug Administration for emergency use, while one has been authorised by the European Medicines Agency (EMA) for use in the European Union (EU; Table 2; FDA, 2021; European Medicines Agency, 2022). The mRNA and vector-based vaccine platforms were used not only to rapidly develop vaccines, but also to produce it at an unprecedented pace. As vaccine production was previously a major limiting step for vaccine distribution and as these platforms facilitate a rapid response to novel pathogens, we argue that these constitute an important innovative means of pharmaceutical intervention to combat future pandemics.

**Table 2:** Comparison of the COVID-19 vaccines approved by FDA and EMA (FDA, 2021; CDC, 2021c; European Medicines Agency, 2022).

Vaccines	Pfizer-BioNTech	Moderna	Janssen	Vaxzevria (previously AstraZeneca)
Date of approval	11 December 2020 (FDA)	18 December 2020 (FDA)	27 February 2021 (FDA)	29 January 2021 (European Union)
Manufacturer	Pfizer, Inc. BioNTech	ModernaTX, Inc.	Janssen Pharmaceuticals Companies of Johnson & Johnson	AstraZeneca AB
Name	BNT162b2	mRNA-1,273	JNJ-78436735	EMEA/H/C/005675
Type of vaccine	mRNA	mRNA	Viral vector	Viral vector
Number of shots	2 shots, 21 days apart	2 shots, 28 days apart	1 shot	2 shots, 28–84 days apart
Authorized Use	Individuals 16yo and older	Individuals 18yo and older	Individuals 18yo and older	Individuals 18yo and older
Main side effects	Pain at the injection site Tiredness Headache Muscle pain Chills Joint pain Fever	Pain at the injection site Tiredness Headache Muscle pain Chills Joint pain Swollen lymph nodes in the same arm as the injection Nausea and vomiting Fever	Pain at the injection site Headache Fatigue Muscle aches Nausea	Tenderness Pain and bruising at the injection site Headache Tiredness Muscle pain General feeling of being unwell Chills Fever Joint pain Nausea

## Modelling

Epidemiological models have become an increasingly ubiquitous tool to support public health policy making, regarding the mitigation of epidemics. The first mathematical formalization of an epidemiological process is commonly attributed to Kermack and Mckendrick (1991a,b,c). As this mathematical framework was published between 1927 and 1933, it clearly postdates the 1918 influenza pandemic. Yet, given the considerable advances with respect to research on model structures and the current computational capabilities, epidemiological models facilitate the investigation of epidemics in detail and aid epidemiologists to inform public health policy in the process.

To study the impact of mitigation policies, a pertinent epidemiological model structure needs to be defined, which depends on the pathogen, its routes of transmission, ecological aspects (e.g. vector ecology) and the character of the investigated mitigation policy. Furthermore, an important decision with respect to model structure is its granularity. Here we discuss the three main model structures: compartment models, individual-based models and meta-population models.

**Figure 6:**
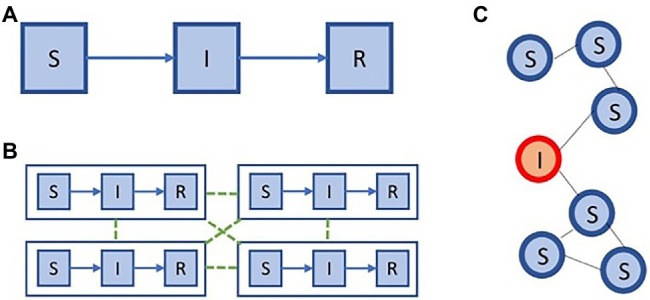
Modelling impact on policy. Two extremes in model space: **(A)** an SIR compartment model, **(C)** and individual-based model. We show a meta population model **(B)**, that is situated between the models in **(A,C)**, in terms of complexity.

Compartment models divide the population into discrete homogeneous states (i.e. compartments) and describe the transition rates from one state to another, *via* which communication between the compartments occurs (Diekmann et al., 2013). For each pathogen, to which a patient can obtain immunity after being infected (e.g. pandemic influenza), we can partition the population in three groups: individuals that are susceptible to infection (i.e. susceptibles), individuals that are infected (i.e. infected), and individuals that recovered and obtained immunity (i.e. recovered; Figure 6 panel **A**). This model, referred to as the SIR model (i.e. abbreviation of Susceptible-Infected-Recovered), was introduced by Kermack and Mckendrick (1991a,b,c). Compartment models can be formalized as a system of ordinary differential equations. While the SIR model is a basic model that assumes homogeneity in the population, it can be extended in various ways to accommodate more complex modelling inquiries.

While compartment models group individuals together based on common properties (e.g. infection or age group), an individual-based model will explicitly represent all individuals and their properties (Diekmann et al., 2013). In this model individuals are connected, either in a static or a dynamic fashion, and the spread of the epidemic is simulated among this network. The most fundamental individual-based model will thus represent each of the individuals and maintain their infection status, i.e. susceptible (S), infected (I), recovered (R). Given this fundamental example, these individuals can be connected using a static network, for example, an Erdos–Rényi random graph, which has a binomial degree distribution. This example is depicted schematically in Figure 6 panel **C**.

On the one hand, compartment models are computationally efficient to evaluate, yet they need to divide the population in coarse compartments. On the other hand, individual-based models allow for more fine-grained modelling, yet require a lot of computation. To balance between these trade-offs, meta-population models are frequently used, especially to enable the modelling of epidemic processes in a spatially explicit context. Meta-population models were first introduced in the context of ecology (Hanski, 1999), to model sub-populations that can be separated geographically. In Figure 6 panel **B**, we show an example of a simple meta-population model with four patches, where each patch consists of an SIR model.

In between these extremes in model space, all kinds of meta-population models can be constructed, as shown in Figure 6. These distinct model structures are important to address different aspects of public health inquiries (Coletti et al., 2021).

While the 1918 influenza pandemic predates the advent of epidemiological models, epidemics of influenza have a predominant place in model literature. On the one hand, the historic 1918 pandemic has been studied extensively using mathematical models (Eggo et al., 2011), and on the other hand, the risk of future pandemics is often explored in an influenza context (Ferguson et al., 2006). The modelling of influenza culminated with the public health modelling efforts related to the 2009 influenza pandemic (Van Kerkhove et al., 2010). As the main body of research focuses on the average reduction in attack rate, in order to reduce the burden with respect to mortality and morbidity, compartment models have been used extensively (Eames et al., 2012). In such models, typically mitigation strategies that are targeted to particular groups of individuals are explored, such as school closures (Wu et al., 2010), antiviral drugs (Lipsitch et al., 2007) and vaccines (Medlock and Galvani, 2009). Furthermore, the spatial spread of the influenza virus and its impact on mitigation policies has been investigated using meta-population models (De et al., 2018) and important advances in the context of large-scale individual-based models have been achieved to study pandemic influenza mitigation (Germann et al., 2006; Chao et al., 2010).

For HIV, compartment models have been used early in the pandemic to understand and estimate the incidence based on observed diagnoses (Sweeting et al., 2005) and continue to be used to evaluate policy options (Kerr et al., 2015). Yet, due to the scale-free nature of the sexual transmission network that enables the transmission of HIV (Liljeros, 2011), and the importance to diagnose individuals early on in their infection (Farnham et al., 2013), interest to use individual-based models for HIV was raised. Through individual-based models, the particularities of scale free sexual networks were investigated (Bakker et al., 2000). Furthermore, they enable the explicit modelling of personal attributes such as partnership properties (Schmid and Kretzschmar, 2012), coinfections with other pathogens that modulate HIV infectiousness (Hendrickx et al., 2021), policies to implement PrEP (LeVasseur et al., 2018), testing and contact tracing strategies (Graw et al., 2012) and virus strains (i.e. HIV subtypes; Ferdinandy et al., 2015).

As a respiratory pathogen, SARS-CoV-2 shares the transmission network and many of the virological properties with influenza viruses. Thus, compartment models with a similar structure than influenza epidemiological models were used to project epidemiological trajectories at the start of the epidemic, to inform policy makers about the expected extent of the burden with respect to hospitalizations and deaths (Kucharski et al., 2020). Yet, early in the pandemic it became clear that the asymptomatic and presymptomatic nature of SARS-CoV-2 infections, combined with differences in disease outcome, needs to be captured in the epidemiological models (Willem et al., 2021).

From a compartment model perspective, this meant that traditional SEIR models (Eames et al., 2012; that is, models that consider four epidemiological states: susceptible, exposed, infected and recovered) required extensions to take into account this additional complexity (Abrams et al., 2021). Compartment models have thus been instrumental in the evaluation of population-wide strategies such as lockdowns, school closures, teleworking and more recently vaccination policies (Prem et al., 2020; Moore et al., 2021). However, in order to study prevention strategies that target the individual, such as contact tracing and mass testing policies, the use of individual-based models provide important insights (Willem et al., 2021). These individual-based models extend the ideas from influenza literature (Chao et al., 2010), but explore novel modelling approaches to respond to particular properties of SARS-CoV-2, including antivirals that modulate the viral load curve of infected individuals (Torneri et al., 2020), understanding the serial and generation intervals under control measures (Torneri et al., 2021), contact tracing telephone apps (Ferretti et al., 2020) and universal testing (Libin et al., 2021). Finally, to study the impact of mobility, meta-population models that were developed in the context of influenza (Dolgin, 2021), were adapted to the COVID-19 context (Coletti et al., 2020; Saldaña and Velasco-Hernández, 2021).

Epidemiological models thus facilitate a framework to make predictions and to study the effect of prevention strategies in simulation. Nevertheless, the development of prevention strategies, which need to fulfil distinct criteria (i.e. prevalence, mortality, morbidity and cost), remains a challenging task. For this reason, the use of machine learning techniques to optimise prevention policies in an epidemiological model constitutes a promising venue for future research. In the context of influenza, the use of reinforcement learning has been shown to learn prevention policies in complex epidemiological models (Libin et al., 2018, 2020). From another perspective, Kerr et al. (2015) use mathematical optimization in combination with compartment models, to optimize HIV mitigation strategies. For COVID-19, both mathematical optimization and reinforcement learning techniques have been used to learn optimal mitigation strategies (Kompella et al., 2020; Richard et al., 2021).

This overview shows that epidemiological models contribute a great amount to the understanding of how pathogens spread, and what can be done to mitigate their spread. A significant part of the power of the public health response to COVID-19, builds on decades of modelling research, but this global emergency also demonstrated some weakness with respect to the state of the reproducibility and availability of the models’ source code (Hazelbag et al., 2020). Therefore, we celebrate the availability of high-quality software code of many important models that are continued to be used to study the ongoing COVID-19 pandemic (Hinch et al., 2021; Kerr et al., 2021; Willem et al., 2021).

## Discussion

In this work, we cover three distinct pandemics and discuss their emergence, zoonotic origin and global spread. Alongside, we compare the public health measures implemented by health authorities to the distinct and similar properties of these pandemics and contextualize it across the eras in which they took place.

During these three pandemics, we witnessed major technical advances regarding the development of pharmaceutical interventions. For SARS-CoV-2, two novel vaccine delivery platforms (i.e. mRNA and vector-based platforms) were used to develop and produce vaccines at an unprecedented pace. These platforms facilitate a swift response to novel pathogens and will be of vital importance to combat future pandemics. The ease of use with which mRNA vaccines can be developed also triggered their use in the context of HIV, for which a vaccine is long due (Aidsmap, 2021). The vaccines targeted at SARS-CoV-2 were developed in less than a year, rendering it an impressive achievement. Nonetheless, it also shows that non-pharmaceutical interventions remain indispensable to react to a pandemic before treatment or vaccines become available. Therefore, studying optimal strategies for non-pharmaceutical interventions remains as crucial as it was for the 1918 influenza pandemic, and it deserves the appropriate attention to prepare for future epidemic calamities. An important concern in the face of the available vaccines, is the continuous evolution of variants, that may affect their effectiveness. This indicates the potential for pan-vaccines, that target a more conserved region in the genome, and could even offer protection for novel pathogens (Wei et al., 2020).

An important determinant in the spread of viruses, is the potential for pre-symptomatic transmission from the infected host to other individuals. This greatly impacts how a pathogen can be controlled, as individuals might be unaware of their infection status before symptoms appear. This is not only a concern for acute respiratory pathogens, such as SARS-CoV-2, but it also plays an important role in the spread of chronic infectious diseases such as HCV and HIV-1, where the presymptomatic phase can last many years. To control pathogens that can be transmitted prior to symptom onset, early diagnosis is key (Fraser et al., 2004).

Recognizing the need for early diagnosis, we could hope to control the next emerging pathogen by means of testing, tow swiftly control the local outbreak, to avoid it to become pandemic. For this to work, we would need to have the capabilities to scale up testing in a short time, to cope with the exponential growth of infections. This could be achieved by advancing diagnostic methods (Kerr et al., 2015) and by maintaining a stock of reagents and diagnostic material that can be flown into the epicentre of the virus outbreak, to enable mass testing to nip the epidemic in the bud.

Eventually, the origin of all novel pathogens lies in a zoonotic jump. Therefore, monitoring ecological niches from which such a jump might originate is of crucial importance. This is especially true for areas where forestial regions are connected to large urban areas. Furthermore, livestock farms remain at risk as farm animals come into close contact with human hosts and are often housed in shelters that facilitate the rapid spread of a novel pathogen. While undoubtedly challenging, we argue that the use of ecological models can be of great use to identify areas that are at risk and warrant close monitoring.

In this work, we address the significant advances related to epidemiological modelling. We are now at the point that flexible individual-based models can quickly be configured to simulate a novel emerging pathogen. Such models have a fine-grained resolution that makes them suitable to model prevention strategies that focus on individuals (i.e. contact tracing, antivirals and isolation) and are crucial during the initial phase of a pandemic. While powerful, such fine-grained models require a detailed understanding of human contacts and mobility. Therefore, we should invest in the collection of data on human contacts and mobility prior to the next epidemic emergency, and study how we can use such datasets to model the impact of social distancing measures on human behaviour. We note that this data is sensitive, and its collection can be prone to ethical and regulatory affairs, which requires the necessary attention. Next to factors that influence human behaviour, individual-based models also require detailed information on properties that are specific to the pathogen under investigation, i.e. the proportion of presymptomatic infection, age factors of the host that modulate virus traits (e.g. virulence, symptoms) and dispersion in infectiousness (i.e. superspreading). Figuring out these factors is challenging while a pandemic unfolds, and therefore, we should prepare by establishing protocols to collect the right data in an optimal way (i.e. transmission networks, genomics, viral load profiles). Such protocols can be optimized by simulating what-if scenarios in individual-based models.

Finally, we note that during the SARS-CoV-2, an unprecedented number of virus sequences was published, *via* the use of next-generation sequencing (NGS) platforms. During the initial phase of an epidemic, sequencing a large proportion of cases can shed light on several virus properties such as the proportion of asymptomatic cases, superspreading effects and properties related to attributes of the host (e.g. age, gender, underlying conditions). In a later phase of the epidemic, sequencing virus genomes allows for monitoring viral diversity, for example, monitoring variants of concerns in the context of SARS-CoV-2. However, it remains to be investigated whether mass sequencing delivers a benefit to a more modest testing policy with a carefully calibrated sampling strategy.

To conclude, we witnessed many advances over this course of these three major pandemics. With all this knowledge, we now have the potential to better prepare for the next pandemic, and perhaps even stop a new pathogen in its tracks before it is able to become a pandemic.

## Author Contributions

MM, PL, and AA: conceptualization. MM and SS: software. MM, MP, AT, PL, and AA: validation and writing—review and editing. MM, VP, MP, SS, PL, and AA: formal analysis. MM, MP, VP, and PL: investigation. MM, SS, and PL: resources. MM, PL, AT, and AA: writing—original draft preparation. MM, MP, VP, PL, and AA: visualization. PL and AA: supervision. AA: project administration and funding acquisition. All authors contributed to the article and approved the submitted version.

## Funding

This study was financed by FCT through the following projects: GHTM-UID/04413/2020, INTEGRIV (PTDC/SAU-INF/31990/2017) and the scholarship PD/BD/135714/2018 and MARVEL (PTDC/SAU-PUB/4018/2021). This project has received funding from the European Union’s Horizon Europe Research and Innovation Programme under grant agreement No. 101046016. This work also received funding from the European Research Council (ERC) under the European Union’s Horizon 2020 research and innovation program (PL and AT: grant number 101003688 – EpiPose project). PL gratefully acknowledges support from the Fonds voor Wetenschappelijk Onderzoek (FWO) *via* postdoctoral fellowship 1242021N and research project G0H0420N, and the Research council of the Vrije Universiteit Brussel (OZR-VUB) *via* grant number OZR3863BOF. This research acknowledges funding from the Flemish Government through the AI Research Program.

## Conflict of Interest

The authors declare that the research was conducted in the absence of any commercial or financial relationships that could be construed as a potential conflict of interest.

## Publisher’s Note

All claims expressed in this article are solely those of the authors and do not necessarily represent those of their affiliated organizations, or those of the publisher, the editors and the reviewers. Any product that may be evaluated in this article, or claim that may be made by its manufacturer, is not guaranteed or endorsed by the publisher.

## Footnotes

^1^ https://pangolin.cog-uk.io
